# Acknowledgement to reviewers

**DOI:** 10.1530/EC-2-A1

**Published:** 2013-11-30

The editorial board wishes to thank the following individuals who have helped to review manuscripts for *Endocrine Connections* during 2013. Their assistance is gratefully acknowledged.

S Azzi

U Baandrup

J Bacchetta

K Badenhoop

A J van Ballegooijen

P Barat

S Behl

C Bergwitz

M Bidlingmaier

L Bonafe

K Brismar

C Buechler

Y Y Chen

D Chia

M S Cooper

S Corbetta

G De Filippo

M F Erdogan

J Faber

K Feingold

E Fliers

P Freitas

S U Gan

N J Gittoes

S Gullu

T K Hansen

R Hardy

J He

V M Howell

A Hubalewska-Dydejczyk

K Javaid

S Kalra

N Krone

Z Laron

D Learoyd

P Lee

L Leenhardt

A Lienhardt-Roussie

S Lightman

P Lips

H Liu

R Ma

V Mohan

D L Morris

J Murray

I G Nad

S Neggers

E F Nemeth

S Nielsen

T Ogawa

O Okosieme

R Opitz

S Orme

A Pandey

N Patel

R Perniola

J Petrie

H Pope

C Posovszky

U Renner

M O Ribeiro

R Sahay

R Salvatori

P J Schaeffer

B C Selvanesan

V Shah

Y shoenfeld

E Sondergaard

K Stochholm

H Sugimoto

G Sunil

M M Swarbrick

M-H Tan

N F Taylor

K A Toulis

M Traub

G T'sjoen

F A Verburg

L Vieira Neto

G P Vinson

V Vishwanathan

P M Yen

Y Zhou

